# Commentary on Demetriou et al. (2018): Methodological and Theoretical Considerations

**DOI:** 10.3390/jintelligence6040053

**Published:** 2018-12-06

**Authors:** Martin Bäckström

**Affiliations:** Department of Psychology, Lund University, 221 00 Lund, Sweden; martin.backstrom@psy.lu.se

The article “Mind-Personality Relations from Childhood to Early Adulthood” attempts to investigate the relation between cognitive ability (GMA) and personality, especially how these two concepts are related during childhood, and whether they may exert influence on each other during development. It is a very ambitious project, using large data samples with wide age ranges and it applies many statistical techniques/methods to test several predictions. The first part of this comment will deal with the issues related to personality theory and measurement, the second part will deal with the statistics and estimations. The comment will not concern theory or measurement of cognitive ability.

The main research question in the paper is that personality influences cognitive development, or vice versa, in the ages between 7 and 20. The data material have the potential to illuminate such questions and some of the results suggest that such influence can be discerned, mostly from personality to cognitive ability. However, this very interesting subject is confused when the authors simultaneously try to answer another, very controversial question related to personality theory. They suggest, in two of the article’s predictions, that personality theoretically can be organized the same way as cognitive ability, i.e., by a hierarchical model with a general factor (GFP) at the top, a second level with two sub-factors (alpha and beta), along with the five-factor model (FFM) below these two levels. This is a topic that has been reviewed several times (e.g., [1]). The GFP model has its proponents [2,3], but it also has its critics [4]. 

Generally, the proponents of GFP suggest that all kinds of correlations between the FFM scales can be used to support the GFP (akin to what is presented in the current paper), whilst the critics have suggested that the correlations are artefacts of the personality measures. The critics of the GFP (e.g., [5,6]) have repeatedly shown that when personality is estimated using a multi-trait multi-method (MTMM) approach, the GFP is not supported (and neither is the alpha and beta). In one study, Danay and Ziegler [7] used an MTMM and found almost no support for a GFP. Other researchers have shown that the self-rated GFP is dependent on how personality is measured. In fact, it is possible to create personality inventories that measure the FFM without any sign of either the GFP or the alpha and beta factors [8]. The source of the GFP has been traced to the evaluativeness of items and it has been suggested that this factor can be controlled for by using multiple raters (self-peer) or by having less evaluative items. The present article uses scales that all have very evaluative items (see [9] for the IPIP-items). There is more to say about the measurement problems, but I would like to emphasize that the design and methods in the studies are neither well suited to estimate a GFP (e.g., no MMTM) nor its eventual relation to GMA.

It is admitted in the paper that there are difficulties related to personality measurement and it is suggested that the kind of personality traits measured are related to the self-concept, i.e., how people perceive themselves in terms of self-worth and likability. Given such a conceptualization, this work is not about personality at all, but about the relation between cognitive ability and the sense of self in different domains. As I see it, the personality measures are treated as if they were de facto measures of the participants’ personality. Let us say that the inventories were just that, measures of personality, then there are still several problems in the article. One problem is that the trajectories of personality vary between the three studies. The most obvious is that extraversion increases in study 2 and decreases in study 3. Psychoticism, which is closely (negatively) related to agreeableness, increases between age 7–9 in study 1, but agreeableness increases in study 2. There is no change in conscientiousness in study 2, but there is in study 3, and there is no discussion of this in the article. I think it is very naïve to interpret higher or lower ratings as corresponding to an increase or decrease in personality traits in these age groups.

One problem mentioned as a limitation in the paper is that the results may have been affected by the children’s lack of self-evaluative ability. I would add a limitation related to language comprehension, as inventories that are validated for adults contain items with words that are difficult for young children to understand. In the older groups, the understanding of words should have developed and some of the changes in relation to personality could be explained by this difference.

The second topic of this comment concerns the statistical estimations and the models that are tested to support the hypotheses of the study. The article includes numerous statistical tests and it is sometimes quite difficult to determine whether the authors exploratorily investigate the data and/or test predictions. In the last study, the replications of the predictions are obscured by the authors use of new statistical methods, e.g., Exploratory Graph Analysis (EGA). It is not feasible to test predictions with EGA, as it is an exploratory technique. In an article concerned with testing predictions, this method does not do the job very well. 

Although there are many statistical methods and tests in the article, there is a lack of information about the variables used. Alpha for the personality inventories are curiously only reported with one figure (there are often five scales used). Moreover, in relation to testing the hypothesis, there is a lack of information, there are no correlation tables, there is no information concerning how the models were defined, the kind of estimations that were used (I assume the Maximum Likelihood), whether the variables were normally distributed, and so on. There is no reference to measurement models, the latent variables are taken for granted, but according to the estimations that I have conducted, there are estimation problems in some of the measurement models, e.g., the GFP model of study 1 and the hierarchical GFP model of study 3. For example, the sub-factors alpha and beta seem to load 1.0 to GFP in many of the models of study 3, which suggests that alpha and beta should have been excluded from the models.

Some of the results, especially the results stemming from the Structural Equation Models could have been discussed more extensively. In some occasions, the results of the estimations seem to be very far from what is theoretically possible. The 1.0 or almost 1.0 coefficients in one model seem to be very difficult to defend theoretically. Since the coefficients estimate relations between latent variables, measured without error, the coefficients can be interpreted as estimating causal relations between constructs. The results suggest that Representation and Inferential power (RIP) predicts the alpha factor almost perfectly, which does not seem defendable. In addition, how is it possible that alpha’s prediction of RIP is almost exactly zero, whilst alpha predicts Processing efficiency (PrEff) almost perfectly? This is especially surprising given that the predictions from Extraversion and Openness of RIP and PrEff are almost exactly the same. I think the cause of these problems is the attempt to estimate personality hierarchically and it may be solved by using a simpler model of the personality structure.

Some of the interpretations of the results are not clearly supported by the estimation. For example, there seems to be more than one kind of GFP. In the last study, there are rather large differences between the observed variables loadings on the GFP. In one model of study 3, the Openness factor and in another the Consciousness factors is the strongest. A perhaps more serious problem is that the paths in the last model do not support a relation between g and GFP. The direct path is negative (at −0.16), and the indirect path over COGN is positive (0.22 × 0.58 = 0.12). Together, they suggest a zero relation. Based on my own calculations, the correlation between the latent GF and the GFP is slightly negative. The following statement is hard to defend if my calculations are correct: “At a higher level, all three studies showed that the relation between g and the GFP was significantly and substantively different from 0 in the models.” When a model is mis-specified, lacks some important relations, or when data is close to multivariate colinear for some observed variables, there is a high risk that the estimated parameter values will be completely off. I fear that this is the case for the models in both study 1 and study 3.

Given both these limitations, related to personality theory and estimation problems, many of the conclusions come across as premature. On the other hand, it is theoretically a very interesting idea, that personality has importance for cognitive development, and vice versa, and this is a field of study that needs more work both theoretically and methodologically. 
